# Circ_0000189 Promotes the Malignancy of Glioma Cells via Regulating miR-192-5p-ZEB2 Axis

**DOI:** 10.1155/2022/2521951

**Published:** 2022-09-19

**Authors:** Jian Yang, Guoqiang Hou, Hongjin Chen, Weilin Chen, Jianwei Ge

**Affiliations:** ^1^Department of Neurosurgery, Renji Hospital, School of Medicine, Shanghai Jiaotong University, Shanghai 200127, China; ^2^Department of Neurosurgery, Xinhua Hospital Affiliated to Shanghai Jiaotong University, Shanghai 200092, China

## Abstract

**Background:**

Some recent studies have reported the role of circular RNAs (circRNAs) in modulating the tumorigenesis of human malignancies. Nevertheless, the expression characteristics, biological functions, and regulatory mechanism of circ_0000189 in glioma are unclear.

**Methods:**

Quantitative real-time polymerase chain reaction (qRT-PCR) was utilized to detect the expression levels of circ_0000189, miR-192-5p, and ZEB2 mRNA in glioma tissues and cells. The association between the expression of circ_0000189 and the clinicopathological indicators and the features of magnetic resonance imaging (MRI) images of glioma patients were analyzed. Western blot was utilized to evaluate ZEB2 expression and epithelial-mesenchymal transition (EMT-)-related proteins (E-cadherin, N-cadherin, as well as Vimentin) in glioma cells. Cell proliferation was assessed employing cell counting kit-8 (CCK-8) and EdU experiments. Flow cytometry was used to detect the apoptotic rate of the cells. Cell migration and invasion were accessed employing Transwell assay. Moreover, dual luciferase reporter gene assay and RNA immunoprecipitation assay were employed to investigate the targeting relationship between miR-192-5p and circ_0000189, miR-192-5p, and ZEB2. Subcutaneous tumorigenesis experiment and lung metastasis experiment in nude mice were conducted to verify the regulatory function of circ_0000189 on the proliferation and metastasis of glioma cells *in vivo*.

**Results:**

circ_0000189 was markedly overexpressed in glioma tissues and cell lines. Its high expression was associated with poor clinical pathological indicators and adverse MRI signs. Gain-of-function experiments and loss-of-function experiments confirmed that circ_0000189 overexpression facilitated the proliferation and migration, as well as invasion of glioma cells, and suppressed apoptosis, and facilitated epithelial-mesenchymal transition (EMT) process. Compared to the control group, knocking down circ_0000189 suppressed the malignant phenotypes of glioma cells *both in vivo* and *in vitro*. Working as a competitive endogenous RNA, circ_0000189 directly targeted miR-192-5p, and repressed its expression, and circ_0000189 positively modulated ZEB2 expression indirectly via repressing miR-192-5p.

**Conclusion:**

circ_0000189 facilitates the progression of glioma by modulating miR-192-5p/ZEB2 axis.

## 1. Introduction

Originating from glial cells, glioma is characterized as the most common malignancy of the nervous system, accounting for approximately 50% of primary intracranial tumors [1]. Although considerable progresses have been made in surgery, chemotherapy, and radiotherapy, as well as immunotherapy, the prognosis of glioma is still far from being satisfactory [2]. Therefore, further investigations on the molecular mechanism of glioma progression are needed to provide novel clues for the diagnosis and treatment of glioma.

Without 5'end cap and a 3'end poly (A) tail, circularRNA (circRNA), a special kind of endogenous noncoding RNA, is formed by reverse splicing, and it is a type of closed-loop RNA molecule [3]. CircRNAs are considered as promising biomarkers and therapeutic targets of cancers [4]. Accumulating studies in recent years have shown that the abnormal expression of circRNAs is associated with the occurrence and development of human malignancies including gliomas [5]. For example, circRIP2 accelerates bladder cancer progression by activating TGF-*β*2/Smad3 signaling [6]. By inducing HIF-1*α* expression, circ-HIPK3 facilitates the proliferation and metastasis of cervical cancer cells [7]. Moreover, circ_0051443 suppresses the malignant biological behavior of liver cancer cells by upregulating BAK1 expression [8]. Circ_0000189 expression is reported to be increased in gastric cancer [9]. However, the expression characteristics, biological functions, and regulatory mechanism of circ_0000189 in glioma are unclear.

miRNA is a kind of noncoding small RNA transcript that modulates the translation of mRNA. It can be fully or partially complementary to the 3′UTR of a specific mRNA and modulate the process of translation. It exerts regulatory functions in biological activities such as cell proliferation, apoptosis, growth, and differentiation, as well as metabolism [10]. Some miRNAs can regulate the expression of a variety of tumor-related genes, and their dysregulation participates in cancer progression [11–13]. Reportedly, by binding to the 3′UTR of E2F7, miR-129-5p suppresses the malignant biological behaviors of rectal adenocarcinoma cells [14]. However, the expression characteristics and the related mechanism of miR-192-5p in gliomas are elusive.

Epithelial-mesenchymal transition (EMT) endows cancer cells with the potential to migrate and invade, and EMT also promotes stemness and resistance to apoptosis and aging and enhances capacity of immune evasion of cancer cells [15–17]. By binding with the promoter of E-cadherin gene, zinc finger E-box binding homeobox 2 (ZEB2) suppresses E-cadherin transcription and induces the EMT of cancer cells, thus facilitating cancer progression [18–21]. However, the upstream mechanism contributing to ZEB2 dysregulation in glioma needs further study.

This study found for the first time that circ_0000189 was markedly overexpressed in gliomas, whose high expression was associated with the adverse clinical pathological indicators and magnetic resonance imaging (MRI) signs. Gain-of-function and loss-of-function experiments suggested that circ_0000189 could promote the malignant biological behaviors of glioma cells. What is more, it was proved that the circ_0000189/miR-192-5p/ZEB2 axis was involved in promoting glioma progression.

## 2. Materials and Methods

### 2.1. Tissue Samples

This study got the approval from the Research Ethics Committee of Renji Hospital, Shanghai Jiaotong University, and the written informed consents of all participating patients were obtained. The tumor tissues of 50 glioma patients diagnosed in Renji Hospital from September 2017 to September 2018 and the corresponding adjacent brain tissues were collected. The clinical and pathological information of the patients was completed (Table 1). The tissue samples were collected during the surgery. After the tissues were resected, the specimens were stored in sterile cryopreservation tubes and immediately frozen in liquid nitrogen for further analysis, and then the expression of circ_000189 was detected by quantitative real-time polymerase chain reaction (qRT-PCR). The MRI images of the corresponding patients were collected before the surgery. The images, T1WI signal and T2WI signal, were judged by two independent radiologists, to evaluate the location of the tumor and the heterogeneity of the signal and peritumoral brain edema.

### 2.2. Cell Culture

Normal human astrocyte cell line (NHA), human normal brain glial cell line (HEB), and human glioma cell lines (A172, U87, LN18, U251, LN229, and U118 cells) were available from China Center for Type Culture Collection (CCTCC, Wuhan, China). The above cells were then cultured in RPMI-1640 medium (Thermo Fisher Scientific, MA, USA) containing 10% fetal bovine serum (FBS) (Gibco, Thermo Fisher Scientific, Shanghai, China) and 100 U/mL penicillin, 100 *μ*g/mL streptomycin (Hyclone, Logan, USA) and cultivated at 37°C in 5% CO_2_. When the cell confluence reached 70-90%, 0.25% trypsin (Hyclone, Logan, USA) was employed for passage.

### 2.3. Cell Transfection

Control plasmid (vector), circ_0000189 overexpression plasmid (circ_0000189), siRNA control (si-vector), siRNAs against circ_0000189 # 1 (si-circ # 1, GAGTAGTATGTTGGTGAGAGT), siRNAs against circ_0000189 # 2 (si-circ # 2, ATGAGTAGTATGTTGGTGAGA), siRNA against ZEB2 (si-ZEB2, GGACACAGGUUCUGAAACA), miRNA control (miR-NC, UUUGUACUACACAAAAGUACUG), miR-192-5p mimics (CUGACCUAUGAAUUGACAGC), and miR-192-5p inhibitors (CUGACCUAUGAAUUGACAGCC) were available from GenePharma (Shanghai, China). A172 and U118 cells were transfected with Lipofectamine®3000 (Invitrogen; Thermo Fisher Scientific, Inc., Shanghai, China). Transfection efficiency was measured by qRT-PCR).

### 2.4. qRT-PCR

Total RNA was extracted from glioma tissue and cells with a RNAiso Plus reagent (Takara, Dalian, China) in accordance with the manufacturer's instruction. The total RNA was reversely transcribed into cDNA with reverse transcriptase kit (Takara, Dalian China). SYBR Green Master Mix (Takara, Dalian, China) was used as a fluorescent indicator, and the reaction of amplification was performed on ABI7300 system (Applied Biosystems, Waltham, MA, UK). GAPDH, hsa_circ_0000284, and U6 were utilized as the endogenous controls in this study. The cycle threshold (Ct) value was employed to calculate the relative gene expressions (formula: the relative expression = 2^−△△Ct^). The primer sequences were listed as follows: circ_0000189 (divergent primer): forward: 5′-TTCTGTATCCGCCTCTGCAT-3′, and reverse: 5′-TGAAAAACTTGTCGCACAGC-3′; circ_0000189 (convergent primer): forward: 5′-CCAAGAACTCAGCACCCTGT-3′, and reverse: 5′-GAGTCTCAATGCTCCCAAGG-3′; hsa_circ_0000284: forward 5′-GTCGGCCAGTCATGTATCAA-3′, and reverse: 5′-ACCAAGACTTGTGAGGCCAT-3′; miR-192-5p: forward: 5′-GGACTTTCTTCATTCACACCG-3′ and reverse: 5′-GACCACTGAGGTTAGAGCCA-3′; ZEB2: forward: 5′-TGAGGATGACGGTATTGC-3′ and reverse: 5′-ATCTCGTTGTTGTGCCAG-3′; GAPDH: forward: 5′-GCACCGTCAAGGCTGAGAAC-3′ and reverse: 5′-TGGTGAAGACGCCAGTGGAGACA-3′; U6: forward: 5′-CTCGCTTCGGCAGCACACA-3′, and reverse: 5′-AACGCTTCACGAATTTGCGT-3′.

### 2.5. Cell Counting Kit-8 (CCK-8) Experiment

The cells in each group during the exponential growth phase were harvested to prepare single cell suspension, and the cell density was modulated after counting the number of cells. After the cells were inoculated into a 96-well plate (1 × 10^3^ cells/well), 10 *μ*L CCK-8 reagent (Beyotime, Shanghai, China) was dripped into each well on days 1, 2, 3, 4, and 5, respectively. 2 h after incubation, the absorbance of each well at 450 nm wavelength was measured employing a Bench-markPlus™ microplate spectrometer. 5 days later, the cell proliferation curves were plotted based on the absorbance values.

### 2.6. EdU Assay

EdU kit (Ruibo, Guangzhou, China) was utilized to evaluate the proliferation of glioma cells. The cells were inoculated into a 24-well plate (with a cover slip inside, 2.5 × 10^5^ cells/well) prior to 24 h of cultivation. EdU solution (working concentration: 50 *μ*mol/L) was added, and the cells were subsequently incubated at 37°C for 2 h prior to the discard of the culture solution. Subsequently, the slide was rinsed with PBS 3 times. Next, paraformaldehyde/glycine was employed for fixation for 30 min. Apollo fluorescent staining reaction solution was utilized for incubation for 30 min in the dark, and the cells were then rinsed twice with methanol. Next, 1 × Hoechst reaction solution was added to stain the nuclei for 30 min, and the cells were subsequently rinsed with PBS 3 times. The total number of cells and EdU-positive cells were randomly counted under the microscope to calculate the percentage of EdU-positive cells.

### 2.7. Scratch Healing Experiment

Glioma cells were inoculated into a 6-well plate (5 × 10^6^/well). When the plate was full of cells, a straight line was drawn with a sterile 200-*μ*L pipette tip to form a direct scratch in the middle of the confluent monolayer cells. Then, the cells and scratch were gently rinsed with serum-free medium for 3 times, and the scratch was observed and photographed under a microscope (0 h). Then, the cells were cultured in serum-free medium for 24 h, and the scratch was observed and photographed under the microscope again (24 h). The healing of the scratch in each group indicated the migration ability of the glioma cells in each group.

### 2.8. Flow Cytometry

AnnexinV-FITC/propidium iodide (PI) double staining method was employed to investigate the apoptosis of glioma cells. About 1 × 10^5^ glioma cells in each group were rinsed with cold PBS two times. Next, 1 × binding buffer was added to resuspend the cells. 5 *μ*L of AnnexinV-FITC staining solution and 5 *μ*L of PI staining solution (Beyotime, Shanghai, China) were subsequently dripped into the cell suspension. Next, the cells were incubated for 15 min in the darkness. Next, 300 *μ*L of binding buffer was added into the cells in each group, and then, the cells were detected with a flow cytometer (Beckman Coulter, Fullerton, CA, USA), and Flowjo V10 software (BD Biosciences, San Jose, CA, USA) was used to measure the apoptotic rates of the cells.

### 2.9. Transwell Assay

Transwell experiment was performed employing Transwell chambers (Millipore, Billerica, USA). The chamber used to measure cell invasion was covered with a layer of Matrigel (Sigma-Aldrich, St Louis, MO, USA) and air-dried overnight. 200 *μ*L of glioma cell suspensions (the cells were resuspended in serum-free medium, 1 × 10^5^ cells/mL) was added to the upper chamber, and 600 *μ*L of medium containing 10% FBS was added into the lower chamber, respectively. Moreover, the upper chamber was then removed after 24 h of incubation, and then, the cells were fixed with 4% paraformaldehyde and stained with 0.1% crystal violet solution. Furthermore, after the cells were rinsed 3 times with PBS, the cells remaining on the upper surface of the membrane were wiped off. After that, the cells were counted under a microscope. For migration assays, Matrigel was not used, and the other steps were as the same as invasion assay.

### 2.10. Western Blotting

Glioma cells were collected, lysed in precooled RIPA lysis buffer (Beyotime Biotechnology, Shanghai, China) for 20 min on ice, and then, the mixtures were centrifuged at high speed for 20 min (13000 r/min, 4°C). The supernatant was harvested after centrifugation, and the protein concentration was quantified employing a bicinchoninic acid (BCA) protein assay kit (Boster Biological Technology, Ltd, China), and the protein samples were boiled at 100°C and denatured for 5 min. Sodium dodecylsulfate polyacrylamide gel electrophoresis (SDS-PAGE) (Beyotime, Beijing, China) was employed for electrophoretic separation, and the protein was subsequently transferred to polyvinylidene fluoride (PVDF) membrane (Millipore, Bedford, MA, USA). The membrane was blocked with 5% skim milk for 2 h at room temperature. Subsequently, diluted primary antibody was added, and the protein was incubated at 4°C overnight. Moreover, the membrane was rinsed with TBST 3 times for 3 min each time. After that, diluted secondary antibody was added, and the membrane was incubated at room temperature for 40 min. Then, the membrane was rinsed with TBST 3 times for 3 min each time. Enhanced chemiluminescent (ECL) kit (Beyotime, Shanghai, China) was used to develop the protein bands. The gray value of each band was analyzed with the software ImageJ, and GAPDH was utilized as endogenous control. The primary antibodies utilized were available from Abcam (Shanghai, China): anti-Bcl-2 antibody (ab32124, 1: 1000), anti-Bax antibody (ab32503, 1: 1000), anti-N-cadherin antibody (ab18203, 1: 1000), anti-E-cadherin antibody (ab194982, 1: 1000), anti-Vimentin antibody (ab92547, 1: 1000), anti-ZEB2 antibody (ab138222, 1: 1000), and anti-GAPDH antibody (ab181602, 1: 2000).

### 2.11. Dual Luciferase Reporter Assay

The binding sites between miR-192-5p and circ_0000189 or 3′-UTR of ZEB2 were predicted through the online bioinformatics analysis database (https://circinteractome.nia.nih.gov/ and https://www.targetscan.org/vert_72/). The corresponding sequences were amplified and inserted into pmiRGLO dual luciferase miRNA target expression vector (Promega, Madison, WI, USA) to establish wild type report vectors pmiRGLO-circ_0000189-WT or pmiRGLO-ZEB2-WT. Subsequently, the mutant (MUT) circ_0000189 or ZEB2 3′-UTR sequences were inserted into the pmiRGLO vector to construct a reporter vector pmiRGLO-circ_0000189-MUT or pmiRGLO-ZEB2-MUT. Additionally, the corresponding reporter vectors and miR-192-5p mimics or control mimics were co-transfected into glioma cells and then incubated for 48 h. Ultimately, the luciferase activity of the cells was accessed employing a dual luciferase reporter assay system (Promega, Madison, WI, USA) according to the manufacturer's protocol.

### 2.12. RNA Immunoprecipitation (RIP)

The Magna RIP RNA-binding protein immunoprecipitation kit (Millipore, Billerica, MA, USA) was used to conduct RIP experiment. Ago2 plasmids were transfected into glioma cells. 1 × 10^7^ cells were subsequently lysed in 100 *μ*L of RIP lysis buffer which contained protease inhibitor cocktail and RNase inhibitors. Next, 5 *μ*g antibody against Ago2 or rabbit IgG coated with magnetic bead was added to incubate with 200 *μ*L of cell lysate at 4°C overnight. Next, after the mixtures were incubated with proteinase K buffer, and the RNA was immunoprecipitated and extracted, reverse transcription was conducted with a Prime-Script RT Master Mix kit (TaKaRa, Dalian, China). Ultimately, the abundance of circ_0000189 and miR-192-5p was assessed with qRT-PCR.

### 2.13. *In Vivo* Experiments

The animal experiments in this study were approved by the Animal Care and Use Committee of Renji Hospital, Shanghai Jiaotong University. Female BALB/c nude mice (5 weeks old) were purchased from Model Animal Research Center of Nanjing University (Nanjing, China). Mice were randomly divided into circ_0000189 knockdown (si-circ) group, circ_0000189 overexpression (circ_0000189) group, ZEB2 knockdown (si-ZEB2) group, and control group (si-con, vector and si-vector) with 10 mice in each group. 1 × 10^7^ U118 cells were inoculated on the back of each mouse, and the tumor volume was measured once a weak. 5 weeks later, the mice were sacrificed, and the tumors were weighted. In the lung metastasis study, 1 × 10^7^ U118 cells were injected into the caudal vein of each mouse, respectively. Two weeks later, mice were sacrificed, and lung metastasis was evaluated by hematoxylin-eosin (HE) staining.

### 2.14. Data Analysis

In this study, all of the experiments were performed in triplicate. The experimental data were then analyzed employing SPSS17.0 statistical software (SPSS Inc., Chicago, IL, USA). The data meeting normal distribution were expressed as mean±standard deviation (*x* ± *s*). The comparison of the data among multiple groups was performed with one-way analysis of variance. LSD method was employed to make the comparison between two groups. Student's *t* test was conducted to make the comparison of data between two groups. *P* < 0.05 was considered to have statistical significance.

## 3. Results

### 3.1. The Characteristics of circ_0000189 Expression in Gliomas and Its Clinical Significance

**Figure 1:**
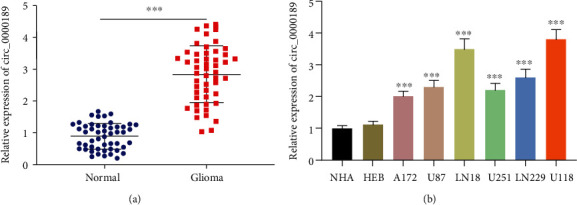
The expression characteristics of circ_0000189 in gliomas. (a) qRT-PCR was utilized to detect circ_0000189 expression in human glioma tissues and adjacent normal tissues (total = 50). (b) Circ_0000189 expression in human glioma cell lines (A172, U87, LN18, U251, LN229, and U118 cells), normal human astrocyte (NHA) cell line, and human normal brain glial cell line (HEB) was investigated by qRT-PCR. ^∗∗^ symbolizes *P* < 0.01, and ^∗∗∗^ symbolizes *P* < 0.001.

The convergent primers and divergent primers were designed, and agarose gel electrophoresis results verified that circ_0000189 had a circular structure (Supplementary Figure 1). The expression levels of circ_0000189 in glioma tissues and adjacent brains tissue were compared with the data of qRT-PCR. The results suggested that compared with that of adjacent brain tissue, the expression of circ_0000189 in glioma tissues was markedly upregulated (Figure 1(a), Supplementary Figure 2). Compared with normal human neuron cell lines NHA and HEB, the expression of circ_0000189 in human glioma cell lines including A172, U87, LN18, U251, LN229, and U118 was also observably upregulated (Figure 1(b)). Additionally, the high expression of circ_0000189 was closely associated with the larger tumor size, higher pathological grade, and nonuniform MRI signal, as well as exacerbation of peritumoral edema (Table 1). These results suggested that circ_0000189 might play a prominent role in the progression of gliomas.

### 3.2. circ_0000189 Facilitated Glioma Cell Proliferation and Repressed Apoptosis

**Figure 2:**
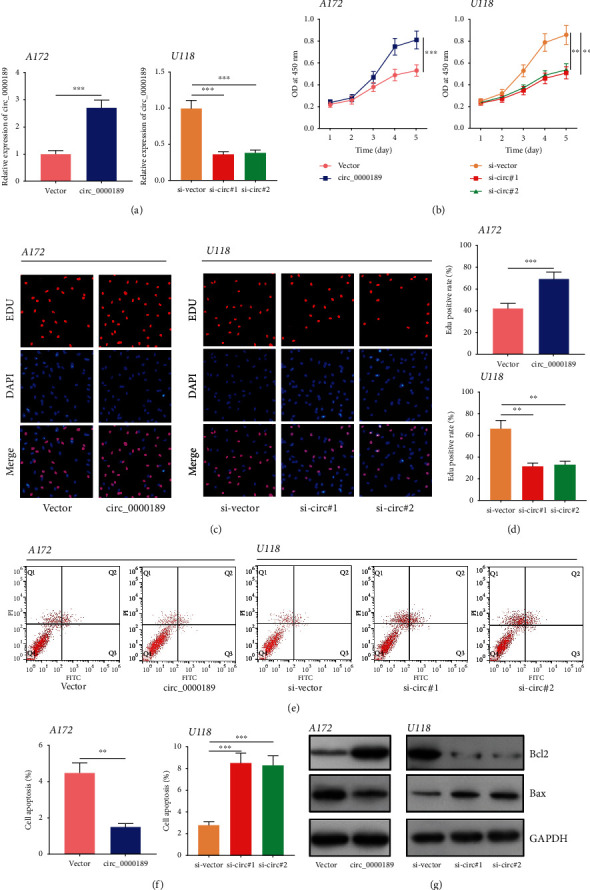
circ_0000189 boosted glioma cell proliferation and impeded apoptosis *in vitro.* (a) A172 cells were transfected with the circ_0000189 overexpression plasmid, and the expression of circ_0000189 in U118 cells was knocked down with siRNA. qRT-PCR was employed to verify the transfection efficiency. (b–d) The proliferation of glioma cells was accessed employing CCK-8 (b) and BrdU (c, d) experiments. (e–f) Apoptosis of the cells in each group was investigated by flow cytometry. (g) Apoptosis-related proteins including Bax and Bcl-2 were detected by Western blot. ^∗∗^ symbolizes *P* < 0.01, and ^∗∗∗^ symbolizes *P* < 0.001.

The above data suggested that the expression of circ_0000189 was the lowest in A172 cells among the glioma cell lines, whereas the expression level of circ_0000189 in U118 cells was the highest. To further study the impact of circ_0000189 in the proliferation and apoptosis of glioma cells, the circ_0000189 overexpression plasmid and si-circ_0000189 were transfected into A172 cells and U118 cells to establish models of circ_0000189 overexpression and knockdown, respectively (Figure 2(a), Supplementary Figure 2). On this basis, CCK-8 experiment, EdU experiment, and flow cytometry were performed. The results suggested that compared with the control group, circ_0000189 overexpression markedly facilitated glioma cell proliferation and suppressed cell apoptosis (Figures 2(b)–2(f)). The expressions of apoptosis markers were then detected employing Western blot. It was revealed that circ_0000189 overexpression promoted Bcl2 expression in A172 cells and suppressed the expression of Bax (Figure 2(g)). Conversely, compared with the control group, knocking down circ_0000189 repressed U118 cell proliferation and induced apoptosis (Figures 2(b)–2(g)). The above data indicated that circ_0000189 participated in facilitating glioma cell proliferation and repressing apoptosis.

### 3.3. Circ_0000189 Facilitated Glioma Cell Movement, Migration, and Invasion, as well as EMT

**Figure 3:**
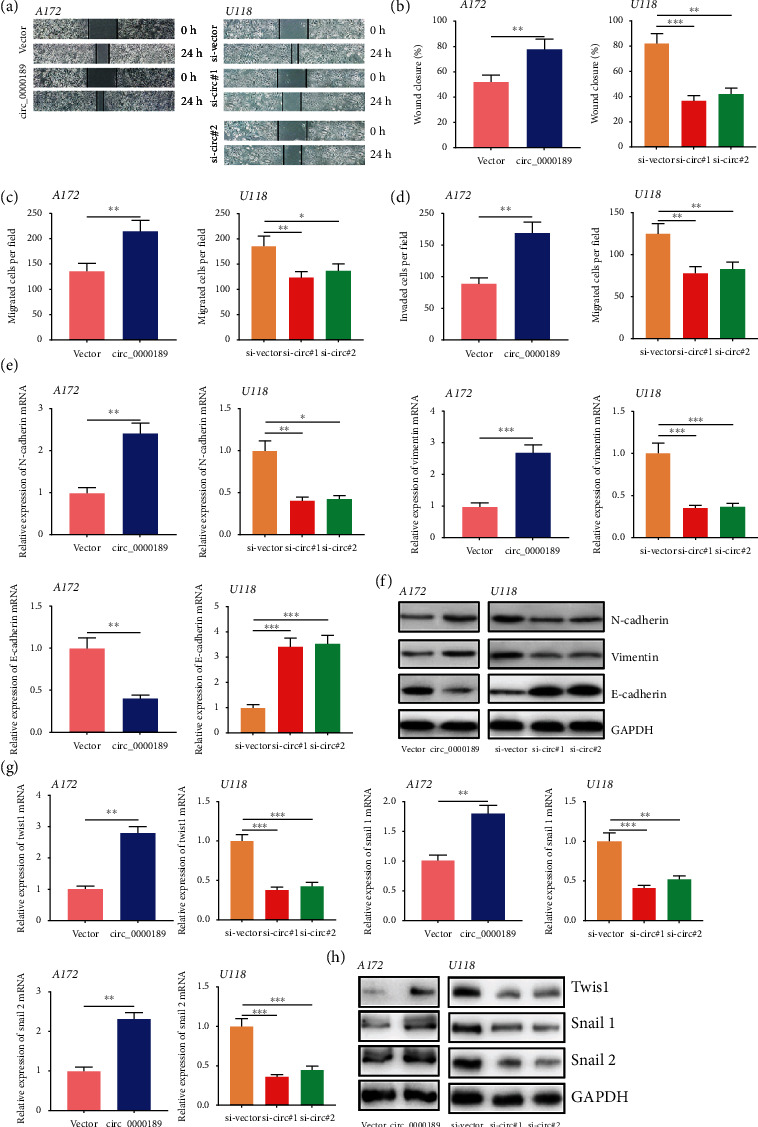
circ_0000189 boosted glioma cell movement, migration, invasion, and EMT. (a–b) Scratch healing experiments were utilized to evaluate the motility of glioma cells. (c–d) Migration and invasion of glioma cells were examined by Transwell experiments. (e–h) EMT-related indicators including N-cadherin, Vimentin, E-cadherin, Twist1, Snail 1, and Snail 2 were detected by qRT-PCR (e and g) and Western blot (f and h). ^∗^ symbolizes *P* < 0.05, ^∗∗^ symbolizes *P* < 0.01, and ^∗∗∗^ symbolizes *P* < 0.001.

The invasive growth of glioma contributes to the unfavorable prognosis of the patients. Then, the motility, migration, and invasion of glioma cells were investigated through scratch healing experiment and Transwell experiment. It was revealed that compared with the control group, circ_0000189 overexpression markedly facilitated A172 cell movement and migration, as well as invasion (Figures 3(a)–3(d)). Epithelial mesenchymal transformation (EMT) is a biological process in which differentiated epithelial cells lost epithelial characteristics and acquired mesenchymal cell characteristics. qRT-PCR and Western blot were utilized to assess EMT-related indicators, including N-cadherin, Vimentin, E-cadherin, Twist1, Snail 1, and Snail 2. The results illustrated that circ_0000189 overexpression markedly facilitated the expressions of N-cadherin, Vimentin, Twist1, Snail 1, and Snail 2 in A172 cells and repressed E-cadherin expression (Figures 3(e)–3(h)). On the contrary, knocking down circ_0000189 attenuated the motility, migration, and invasion ability of glioma cells and inhibited EMT (Figures 3(a)–3(h)).

### 3.4. circ_0000189 Directly Targeted miR-192-5p

**Figure 4:**
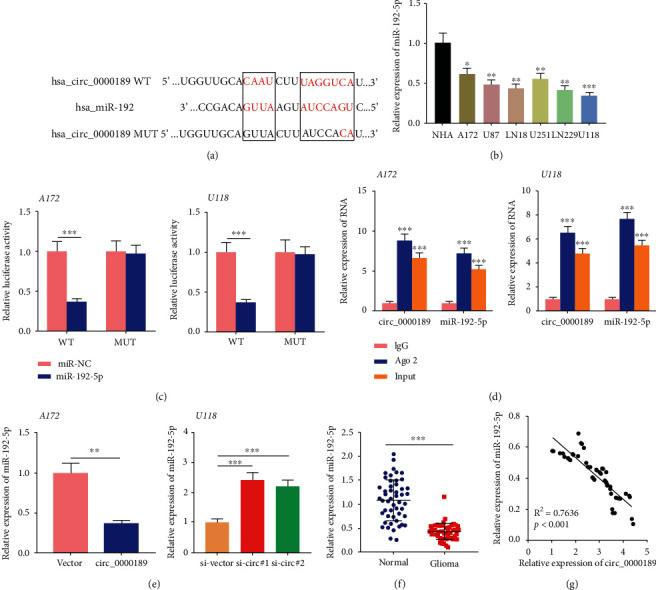
miR-192-5p was a target of circ_0000189 in gliomas. (a) Bioinformatics predicted the binding site between circ_0000189 and miR-192-5p. (b) qRT-PCR was utilized to explore miR-192-5p expression in the normal human astrocyte (NHA) cell line and human glioma cell lines (A172, U87, LN18, U251, and LN229, as well as U118 cells). (c) Dual luciferase report assay was performed to verify the predicted binding site between miR-192-5p and circ_0000189. (d) RIP experiment was performed to verify the direct interaction between circ_0000189 and miR-192-5p. (e) After upregulating the expression of circ_0000189 in A172 cells and knocking down the expression of circ_0000189 in U118 cells, qRT-PCR was utilized to investigate miR-192-5p expression. (f) miR-192-5p expression in normal tissues and glioma tissues was detected by qRT-PCR. (g) Analysis of the correlation between circ_0000189 expressions and miR-192-5p expression in glioma tissues. ^∗^ symbolizes *P* < 0.05, ^∗∗^ symbolizes *P* < 0.01, and ^∗∗∗^ symbolizes *P* < 0.001.

Circinteractome database was employed to predict the miRNAs that could probably interact with circ_0000189, and a potential binding site was found between circ_0000189 and miR-192-5p (Figure 3(a)). The prediction scores of miR-215 and miR-607 were also high; however, miR-215 and miR-607 were not dysregulated in glioma tissues (Supplementary Figures 3 and 4). qRT-PCR was then utilized to detect miR-192-5p expression in NHA cells and glioma cells, the results of which showed that compared with NHA cells, miR-192-5p expression was markedly downregulated in human glioma cell lines A172, U87, LN18, U251, LN229, and U118 (Figure 4(b)). Dual luciferase reporter experiment confirmed that miR-192-5p mimics markedly repressed the luciferase activity of wild type circ_0000189 reporter, but could not significantly weaken the luciferase activity of the mutant circ_0000189 reporter (Figure 4(c)). Furthermore, RIP experiment confirmed that compared with control IgG, circ_0000189 and miR-192-5p were enriched in Ago2-containing microribonucleoproteins, suggesting that miR-192-5p and circ_0000189 were directly interacted with each other (Figure 4(d)). It was subsequently revealed that circ_0000189 overexpression in A172 cells markedly suppressed miR-192-5p expression, whereas the knockdown of circ_0000189 in U118 cells increased miR-192-5p expression (Figure 4(e)). In addition, qRT-PCR showed that compared with normal tissues, miR-192-5p expression was notably downregulated in glioma tissues (Figure 4(f)). The association between circ_0000189 and miR-192-5p expressions in 50 patients was then analyzed, and it was observed that they were strongly and negatively correlated (Figure 4(g)). These results indicated that circ_0000189 acted as a molecular sponge for miR-192-5p.

### 3.5. miR-192-5p Repressed Glioma Cell Proliferation and Facilitated Apoptosis

**Figure 5:**
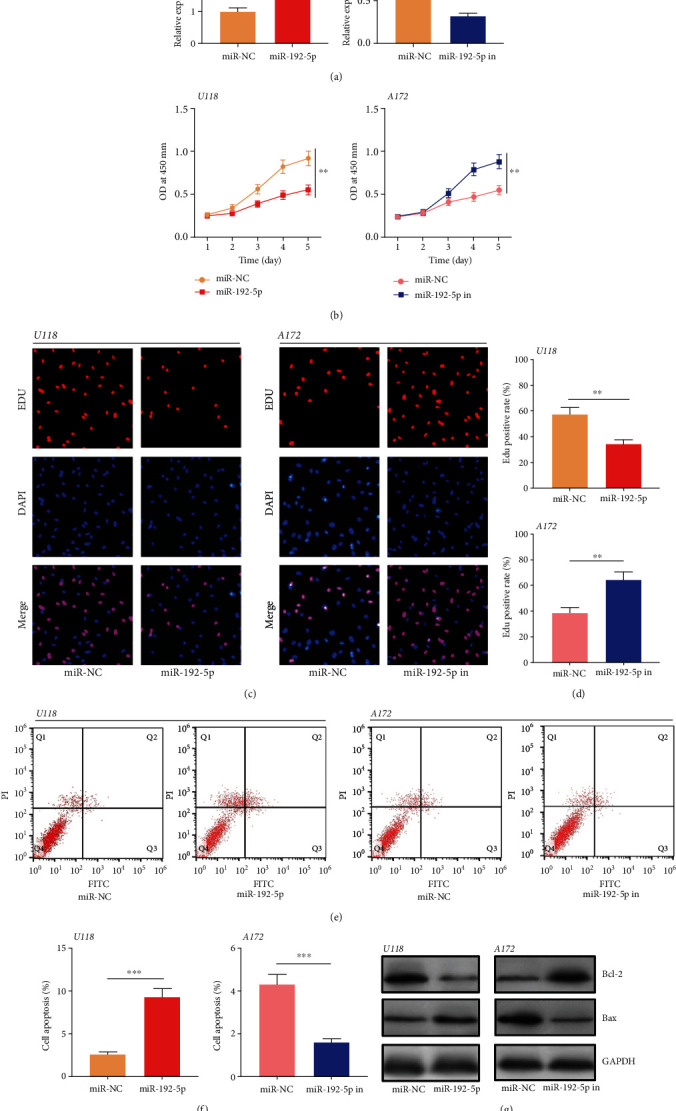
miR-192-5p impeded glioma proliferation and facilitated apoptosis. (a) miR-192-5p mimics were transfected into U118 cells, and miR-192-5p inhibitors were transfected into A172 cells, and qRT-PCR was employed to explore the transfection efficiency. (b–d) Cell proliferation was measured employing CCK-8 (b) and EdU (c, d) experiments. (e–f) Apoptosis of glioma cells was investigated by flow cytometry. (g) Apoptosis-related proteins including Bax and Bcl-2 were detected by Western blot. ^∗^ symbolizes *P* < 0.05, ^∗∗^ symbolizes *P* < 0.01, and ^∗∗∗^ symbolizes *P* < 0.001.

The expression of miR-192-5p was the lowest in U118 cells among the glioma cells, while A172 cells had the highest expression (Figure 4(b)). To further study the biological functions of miR-192-5p in glioma, U118 cells and A172 cells were transfected with miR-192-5p mimics and miR-192-5p inhibitors, respectively (Figure 5(a)). CCK-8 experiments, EdU experiments, and flow cytometry were then performed. The results showed that compared with the control group, the transfection of miR-192-5p mimics repressed the proliferation and promoted the apoptosis of U118 cells (Figures 5(b)–5(f)). Western blot result proved that miR-192-5p mimics promoted Bax expression and impeded Bcl-2 expression in U118 cells (Figure 5(g)). On the contrary, compared to the control group, transfection of miR-192-5p inhibitors promoted the proliferation of A172 cells and repressed apoptosis. The above data suggested that miR-192-5p functioned as a tumor suppressor in glioma.

### 3.6. miR-192-5p Impeded Glioma Cell Movement, Migration, and Invasion, as well as EMT

**Figure 6:**
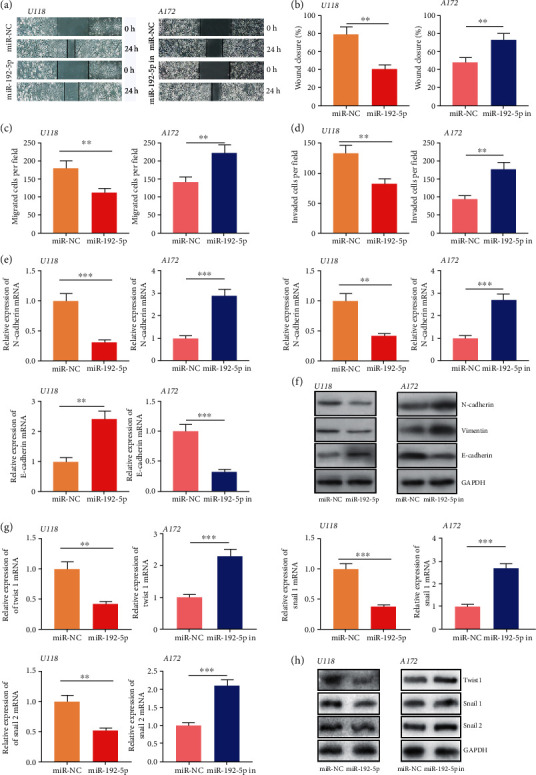
miR-192-5p repressed glioma cell movement, migration, invasion, and EMT. (a–b) Scratch healing experiments were employed to explore glioma cell motility. (c–d) The migration (c) and invasion (d) of glioma cells were detected by Transwell experiment. (e–h) EMT-related indicators including N-cadherin, Vimentin, E-cadherin, Twist1, Snail 1, and Snail 2 and were detected by qRT-PCR (e and g) and Western blot (f and h), respectively. ^∗^ symbolizes *P* < 0.05, ^∗∗^ symbolizes *P* < 0.01, and ^∗∗∗^ symbolizes *P* < 0.001.

Furthermore, the abilities of glioma cells to move, migrate, and invade were further evaluated through scratch healing experiments and Transwell experiments. The results indicated that compared to the control group, overexpressed miR-192-5p markedly impeded the movement and migration, as well as invasion of U118 cells (Figures 6(a)–6(d)). Subsequently, qRT-PCR and Western blot results illustrated that compared to control group, miR-192-5p overexpression significantly impeded the expressions of N-cadherin, Vimentin, Twist1, Snail 1, and Snail 2 in U118 cells and induced the expression of E-cadherin (Figures 6(e)–6(h)). On the other hand, compared to control group, miR-192-5p inhibitors facilitated the motility, migration, invasion, and EMT of glioma cells (Figures 6(a)–6(h)). These experiments illustrated that miR-192-5p impeded glioma cell movement, migration, and invasion, as well as EMT.

### 3.7. circ_0000189 Facilitated Malignant Phenotype of Glioma Cells by Adsorbing miR-192-5p

**Figure 7:**
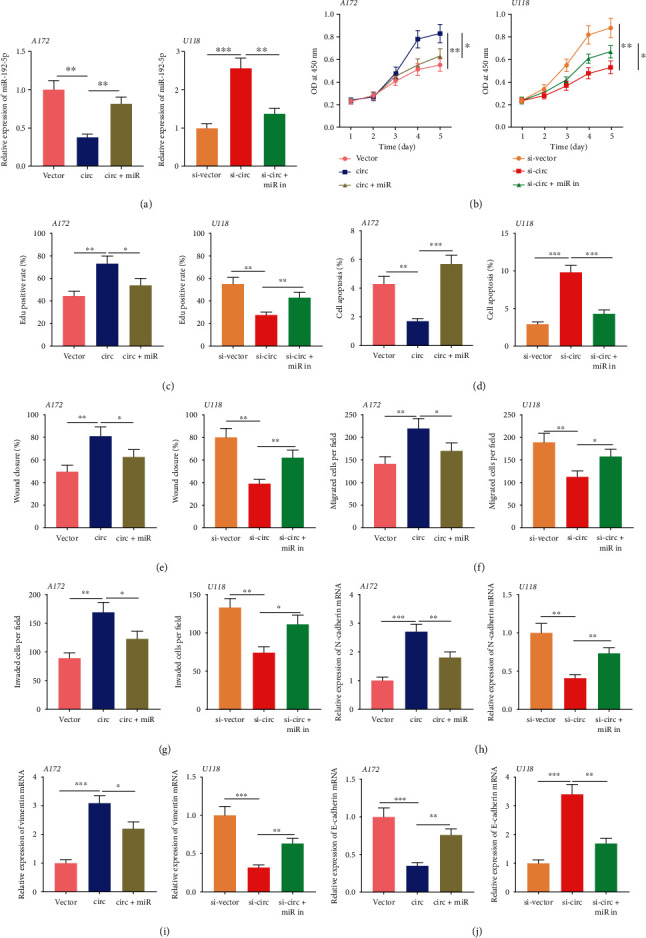
circ_0000189/miR-192-5p axis participated in regulating malignant behaviors of glioma cells. (a) miR-NC or miR-192-5p mimics were transfected into A172 cells with circ_0000189 overexpression. miR-NC or miR-192-5p inhibitors were transfected into U118 cells with circ_0000189 knockdown. Then, qRT-PCR was employed to detect miR-192-5p expression in glioma cells. (b–c) CCK-8 and BrdU experiments were employed to evaluate the proliferation of glioma cells. (d) Apoptosis of glioma cells was assessed by flow cytometry. (e) The scratch healing experiment was employed to evaluate the motility of glioma cells. (f–g) Transwell experiments were employed to examine glioma cell migration (f) and invasion (g). (h–j) EMT-related indicators such as N-cadherin, Vimentin, and E-cadherin were detected by qRT-PCR. ^∗^ symbolizes *P* < 0.05, ^∗∗^ symbolizes *P* < 0.01, and ^∗∗∗^ symbolizes *P* < 0.001.

To explore the functions of the circ_0000189/miR-192-5p axis in gliomas, miR-192-5p mimics were transfected into in A172 cells with circ_0000189 overexpression, and miR-192-5p inhibitors were subsequently transfected into U118 cells with circ_0000189 knockdown (Figure 7(a)). It was revealed that the promotion of the malignant biological behaviors of A172 cells caused by circ_0000189 overexpression was partially reversed by the upregulation of miR-192-5p (Figures 7(b)–7(j)). Conversely, the inhibition of the malignant biological behaviors of U118 cells caused by circ_0000189 knockdown was partially abolished by the co-transfection of miR-192-5p inhibitors (Figures 7(b)–7(j)). These data suggested that circ_0000189 took part in the progression of glioma by adsorbing miR-192-5p.

### 3.8. miR-192-5p Directly Targeted ZEB2

**Figure 8:**
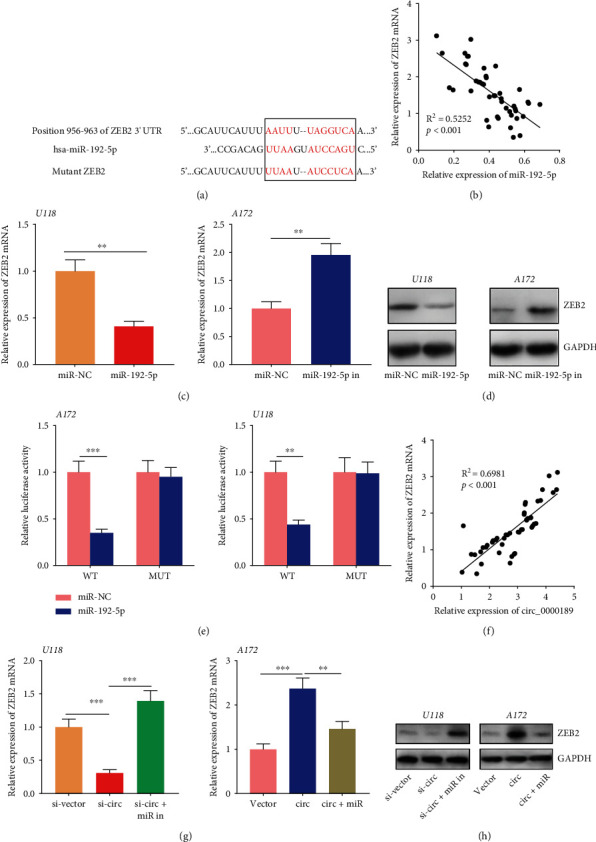
miR-192-5p paired with 3′UTR of ZEB2. (a) Bioinformatics analysis predicted the binding site between miR-192-5p and the 3′UTR of ZEB2. (b) The correlation between the miR-192-5p expression and ZEB2 mRNA expression in glioma tissues. (c, d) qRT-PCR (c) and Western blot (d) were performed to detect ZEB2 expression in glioma cells after transfection of miR-192-5p mimics or inhibitors. (e) Dual luciferase reporter assay indicated that miR-192-5p overexpression suppressed the luciferase activity of wild-type ZEB2 reporter, but did not markedly repress the luciferase activity of mutant ZEB2 reporter. (f) The correlation between circ_0000189 expression and ZEB2 mRNA expression in glioma tissues. (g and h) si-circ_0000189 and miR-192-5p inhibitors were co-transfected into U118 cells, and circ_0000189 overexpression plasmid and miR-192-5p mimics were co-transfected into A172 cells, and the expression of ZEB2 was detected by qRT-PCR (g) and Western blot (h), respectively. ^∗∗^ symbolizes *P* < 0.01, and ^∗∗∗^ symbolizes *P* < 0.001.

TargetScan (http://www.targetscan.org) database was utilized to screen candidate targets for miR-192-5p, and it was revealed that ZEB2 was one of the candidate targets for miR-192-5p (Figure 8(a), Supplementary Table 1). In previous studies, ZEB2 has been recognized as a key biomarker in glioma, which promotes proliferation, migration, and invasion by inducing EMT process [22, 23]. qRT-PCR showed that miR-192-5p expression in the tissues of 50 glioma patients and the expression of ZEB2 mRNA were negatively correlated (Figure 8(b)). qRT-PCR and Western blot showed that miR-192-5p overexpression markedly repressed ZEB2 expression at both mRNA and protein levels, whereas downregulating miR-192-5p increased ZEB2 expression in glioma cells (Figures 8(c) and 8(d)). Dual luciferase reporter experiment was conducted to assess whether miR-192-5p could bind to 3′-UTR of ZEB2. The result showed that miR-192-5p mimic markedly weakened the luciferase activity of wild-type ZEB2 reporter, whereas no obvious change in the luciferase activity of mutant ZEB2 reporter induced by miR-192-5p was found (Figure 8(e)). Additionally, the expressions of circ_0000189 and ZEB2 mRNA were positively correlated in glioma tissues (Figure 8(f)). What is more, qRT-PCR and Western blot suggested that knockdown of circ_0000189 repressed the expression of ZEB2 in glioma cells, and co-transfection of miR-192-5p inhibitors reversed this change; overexpression of circ_0000189 promoted the expression of ZEB2 in glioma cells, while co-transfection of miR-192-5p mimics abrogated this effect (Figures 8(g) and 8(h)). These results indicated that miR-192-5p directly targeted ZEB2, and circ_0000189 could positively regulate the expression of ZEB2 via repressing miR-192-5p in glioma cells.

### 3.9. circ_0000189 and ZEB2 Affected Glioma Proliferation and Metastasis *In Vivo*

**Figure 9:**
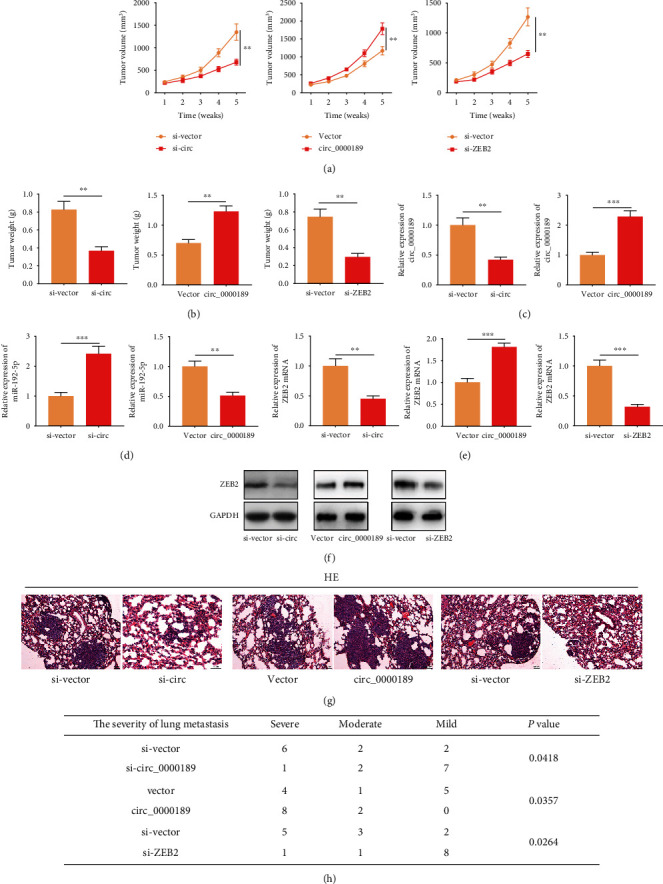
circ_0000189 and ZEB2 affected tumor growth and metastasis *in vivo.* (a–b) The volume (a) and weight (b) of tumor after circ_0000189 knockdown or overexpression, or ZEB2 knockdown *in vivo* was tested. (c–e) qRT-PCR was employed to explore the expressions of circ_0000189 (c), miR-192-5p (d), and ZEB2 mRNA (e) in tumor tissues of the mice in each group. (f) ZEB2 expression in tumor tissues was detected by Western blot. (g–h) HE staining was employed to detect the lung metastasis of the mice in each group. ^∗^ symbolizes *P* < 0.05, ^∗∗^ symbolizes *P* < 0.01, and ^∗∗∗^ symbolizes *P* < 0.001.

In subcutaneous xenotransplanted tumor model, compared with the control group, the tumor volume of the mice was markedly decreased after knockdown of circ_0000189 or ZEB2, but the tumor volume was increased after circ_0000189 overexpression (Figure 9(a), Supplementary Figure 1). Additionally, the average tumor weight in the si-circ_0000189 or si-ZEB2 group was observably lower than that in the si-vector group; however, circ_0000189 overexpression increased the tumor weight (Figure 9(b)). As shown, compared with si-vector group, the expressions of circ_0000189 and ZEB2 mRNA in tumor tissues of si-circ_0000189 group were markedly downregulated, and miR-192-5p expression was observably increased, and the opposite results were found in the circ_0000189 overexpression group (Figures 9(c)–9(e)). Western blot showed that compared with the control group, ZEB2 expression was declined in tumor tissues after circ_0000189 or ZEB2 was knocked down, but ZEB2 expression was increased in tumor tissues after circ_0000189 overexpression (Figure 9(f)). Moreover, lung metastasis model was established to evaluate the aggressiveness of glioma cells. It was demonstrated that knockdown of circ_0000189 or ZEB2 weakened the lung metastasis of glioma cells, but circ_0000189 overexpression promoted the lung metastasis (Figures 9(g) and 9(h)). These *in vivo* data further implied that circ_0000189 was a regulator of glioma progression.

## 4. Discussion

CircRNA is conserved across species and expressed with temporal and spatial specificity [5, 24]. Most circRNAs are with covalently closed circular structure, which are produced by alternative splicing of exons of protein-coding genes, and they are not sensitive to nucleases; therefore, circRNAs are more stable than linear RNAs [25]. In recent years, circRNA becomes increasingly important in the field of cancer research, and it is associated with the pathogenesis of multiple human cancers including gliomas [26–28]. For example, the expression of circHIPK3 in glioma tissue is markedly upregulated, and its high expression is observably to be correlated with the poor prognosis of the patients; circHIPK3 facilitates the proliferation and invasion of glioma cells by modulating IGF2BP3 expression [27]. For the first time, the present study found that the expression of circ_0000189 in glioma tumor tissues and cells was increased significantly, and its high expression was associated with larger tumor volume, higher clinical grade, and adverse MRI image characteristics. These results implied that circ_0000189 could probably be an indicator of glioma progression. Additionally, compared with the control group, circ_0000189 overexpression could markedly facilitate the malignant biological behaviors of glioma cells, whereas the opposite effect was found after knocking down circ_0000189. Collectively, these experiments suggested that circ_0000189 played a carcinogenic role in gliomas, and it could be regarded as a potential therapeutic target.

miRNA, a class of small RNA with a length of 16-26 nucleotides, negatively regulates gene expression at the translation level by pairing with target mRNA [29]. miRNAs take part in regulating various biological processes, including embryonic development, cell proliferation, apoptosis, and even tumorigenesis and progression [30]. It is reported that the expression of 30% genes is modulated by miRNAs, and in cancer biology, miRNAs can function as proto-oncogenes or tumor suppressors [11, 31]. Exploring the relationship between miRNA and the biological behaviors of tumor cells can offer new clues for early diagnosis and monitoring progression, as well as prognosis evaluation. MiR-192-5p participates in regulating the progression of many tumors. For example, during bone metastasis in lung cancer, miR-192-5p impedes cell proliferation and migration, as well as invasion by targeting the inhibition of TRIM44 expression [32]. miR-192-5p represses bladder cell growth by repressing YY1 [33]. In colonic cancer, miR-192-5p exerts a tumor suppressive impact by regulating PI3K/Akt signaling [34]. The current study showed that miR-192-5p expression was downregulated in glioma tissues and cell lines; *in vitro* experiments confirmed that miR-192-5p repressed the malignant biological behaviors of glioma cells. For the first time, we proved that miR-192-5p was a tumor suppressor in glioma.

CircRNA can adsorb miRNA via miRNA response element and indirectly regulates gene expression [35, 36]. For example, circPTN promotes the stemness of glioma cells by adsorbing miR-145-5p/miR-330-5p [37]. Circ_0014359 facilitates glioma progression by modulating the miR-153/PI3K axis [38]. The binding site between circ_0000189 and miR-192-5p was predicted by bioinformatics analysis in this study. Dual luciferase reporter gene experiment, RIP experiment, and qRT-PCR confirmed that circ_0000189 could adsorb miR-192-5p and negatively modulate its expression. Additionally, it was found that miR-192-5p mimics reversed the promoting effects of circ_0000189 overexpression on the malignant phenotypes of glioma cells, whereas malignant biological behaviors of glioma cells inhibited by circ_0000189 knockdown were partially alleviated by the co-transfection of miR-192-5p inhibitors. It is concluded that circ_0000189 took part in the regulation of glioma cell proliferation, apoptosis, and migration, as well as invasion by adsorbing miR-192-5p expression.

ZEB2 is a zinc finger structural transcription factor and belongs to the ZEB family. It regulates various biological activities such as cell growth, apoptosis, and inflammatory response [39]. It is found that ZEB2 can bind to CACCT (G) of the E2 box on the promoter of E-cadherin-encoding genes, suppress the transcription of E-cadherin, induce the EMT of cells, and enhance cell metastasis [40]. Additionally, ZEB2 is reported to promote the transcription of vimentin and N-cadherin during EMT, which is required for pseudopodium formation and cell motility [41, 42]. Additionally, previous studies have indicated that ZEB2 is regulated by multiple miRNAs. For example, miR-1179 inhibits liver cancer metastasis by suppressing ZEB2 expression [43]. miR-769-3p and miR-590-3p repress glioma progression by repressing ZEB2 [44, 45]. In the present work, it was confirmed that miR-192-5p and the 3′UTR of ZEB2 had a binding site. Overexpression of miR-192-5p could repress ZEB2 expression in glioma cells, while inhibiting miR-192-5p caused upregulation of ZEB2 expression. Additionally, we proved that circ_0000189/miR-192-5p regulated ZEB2 expression. Our results proposed a novel competitive endogenous RNA (ceRNA) network composed of circi_0000189, miR-192-5p, and ZEB2, which was involved in glioma progression.

To sum up, this study for the first time reports the high expression of circ_0000189 in glioma tissues and cell lines, and it is also associated with the adverse clinical characteristics of glioma patients. Moreover, circ_0000189 facilitates cell proliferation, migration, and invasion, as well as EMT process, and represses apoptosis through the miR-192-5p/ZEB2 axis, suggesting that it is a valuable candidate target for glioma treatment.

## Figures and Tables

**Table 1:** Correlations between circ_0000189 expression and clinical characteristics in glioma patients.

Pathological indicators	Number of patients	Relative expression of circ_0000189		*P* value
		High	Low	
All cases	50	25	25	
Age (years)				
<30	17	11	6	0.136
≥30	33	14	19	
Gender				
Male	24	13	11	0.571
Female	26	12	14	
Tumor diameter				
< 3 cm	23	8	15	0.047
> 3 cm	27	17	10	
Pathological type				
Astrocytoma	24	14	10	0.499
Oligodendroglioma	18	8	10	
Others	8	3	5	
Pathological grade				
I-II	22	7	15	0.023
III-IV	28	18	10	
Tumor signal				
Uniform	24	8	16	0.024
Non-uniform	26	17	9	
Peritumoral edema				
Mild	20	6	14	0.021
Moderate to severe	30	19	11	
WHO grades				
I-II	28	11	17	0.087
III-IV	22	14	8	

## Data Availability

The data used to support the findings of this study are available from the corresponding author upon request.

## Supplementary Materials

Supplementary Materials Supplementary Figure 1. The structure characteristics of circ_0000189. (a) The formation, convergent and divergent primers of circ_0000189. (b) The PCR products of circ_0000189 were tested by gel electrophoresis. Divergent primers amplified circ_0000189 in cDNA but not genomic DNA (gDNA). Supplementary Figure 2. The expression of circ_0000189 in glioma tissues and cells. (a) qRT-PCR was utilized to detect circ_0000189 expression in human glioma tissues and adjacent normal tissues (total =50), with hsa_circ_0000284 as the endogenous control. (b) Circ_0000189 expression in human glioma cell lines (A172, U87, LN18, U251, LN229, and U118 cells) and normal human astrocyte (NHA) cell line was investigated by qRT-PCR, with hsa_circ_0000284 as the endogenous control. (c) qRT-PCR was employed to test circ_0000189 expression after circ_0000189 knockdown or overexpression in glioma cells, with hsa_circ_0000284 as the endogenous control. (d) qRT-PCR was employed to test circ_0000189 expression after circ_0000189 knockdown or overexpression in the tumor tissues of mice, with hsa_circ_0000284 as the endogenous control. ^∗∗^ symbolizes *P* < 0.01, and ^∗∗∗^ symbolizes *P* < 0.001. Hsa_circ_0000284 were utilized as the endogenous controls. Supplementary Figure 3. The potential targets of circ_0000189 predicted by Circinteractome database. Supplementary Figure 4. The expressions of miR-215 and miR-607 in normal tissues and glioma tissues was detected by qRT-PCR. Supplementary Table 1. The potential targets of miR-192-5p predicted by TargetScan database.
