# Supplementary material for: Circ_0000189 Promotes the Malignancy of Glioma Cells via Regulating miR-192-5p-ZEB2 Axis
Source: Oxid Med Cell Longev. 2022 Sep 19;2022:2521951. doi: 10.1155/2022/2521951 (PMC9526621; doi:10.1155/2022/2521951)
Supplement: Supplementary Materials — Supplementary Figure 1. The structure characteristics of circ_0000189. (a) The formation, convergent and divergent primers of circ_0000189. (b) The PCR products of circ_0000189 were tested by gel electrophoresis. Divergent primers amplified circ_0000189 in cDNA but not genomic DNA (gDNA). Supplementary Figure 2. The expression of circ_0000189 in glioma tissues and cells. (a) qRT-PCR was utilized to detect circ_0000189 expression in human glioma tissues and adjacent normal tissues (total =50), with hsa_circ_0000284 as the endogenous control. (b) Circ_0000189 expression in human glioma cell lines (A172, U87, LN18, U251, LN229, and U118 cells) and normal human astrocyte (NHA) cell line was investigated by qRT-PCR, with hsa_circ_0000284 as the endogenous control. (c) qRT-PCR was employed to test circ_0000189 expression after circ_0000189 knockdown or overexpression in glioma cells, with hsa_circ_0000284 as the endogenous control. (d) qRT-PCR was employed to test circ_0000189 expression after circ_0000189 knockdown or overexpression in the tumor tissues of mice, with hsa_circ_0000284 as the endogenous control. ∗∗ symbolizes P < 0.01, and ∗∗∗ symbolizes P < 0.001. Hsa_circ_0000284 were utilized as the endogenous controls. Supplementary Figure 3. The potential targets of circ_0000189 predicted by Circinteractome database. Supplementary Figure 4. The expressions of miR-215 and miR-607 in normal tissues and glioma tissues was detected by qRT-PCR. Supplementary Table 1. The potential targets of miR-192-5p predicted by TargetScan database. [file 2521951.f1.doc]

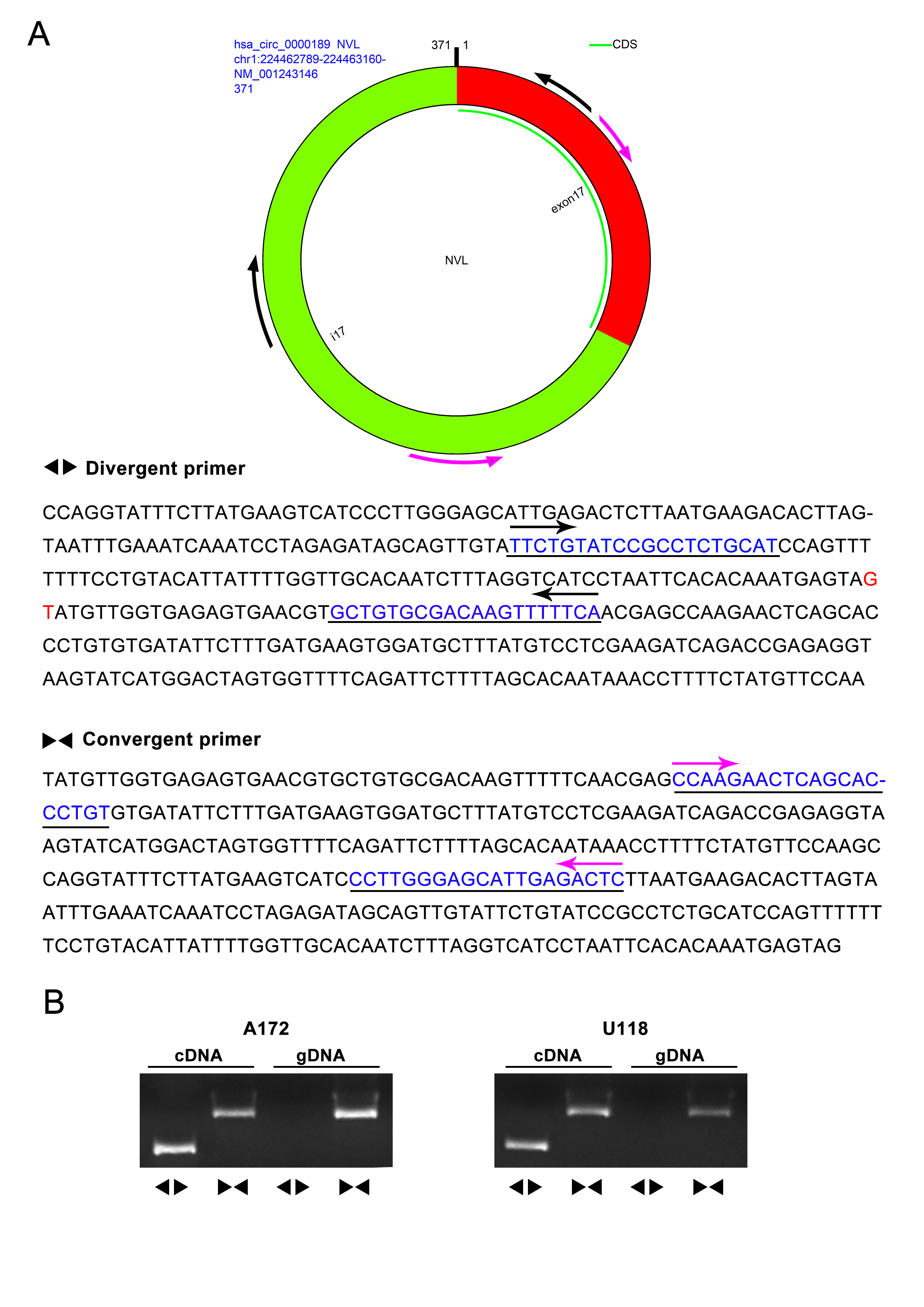


**Supplementary Figure 1. The structure characteristics of circ_0000189.**

(A) The formation, convergent and divergent primers of circ_0000189.

(B) The PCR products of circ_0000189 were tested by gel electrophoresis. Divergent primers amplified circ_0000189 in cDNA but not genomic DNA (gDNA).


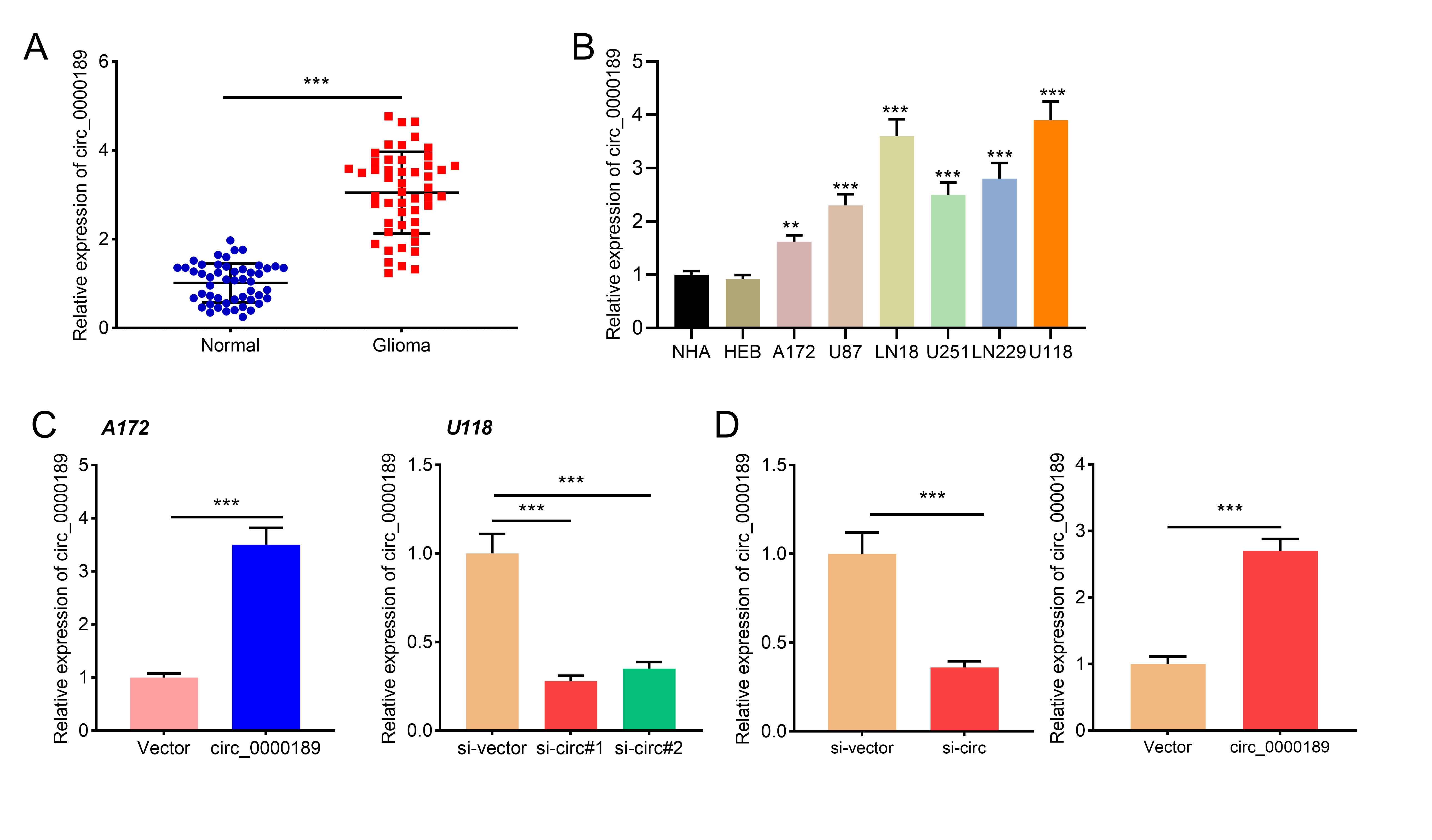


**Supplementary Figure 2. The expression of circ_0000189 in glioma tissues and cells.**

(A) qRT-PCR was utilized to detect circ_0000189 expression in human glioma tissues and adjacent normal tissues (total = 50), with hsa_circ_0000284 as the endogenous control.

(B) Circ_0000189 expression in human glioma cell lines (A172, U87, LN18, U251, LN229, and U118 cells) and normal human astrocyte (NHA) cell line was investigated by qRT-PCR, with hsa_circ_0000284 as the endogenous control.

(C) qRT-PCR was employed to test circ_0000189 expression after circ_0000189 knockdown or overexpression in glioma cells, with hsa_circ_0000284 as the endogenous control.

(D) qRT-PCR was employed to test circ_0000189 expression after circ_0000189 knockdown or overexpression in the tumor tissues of mice, with hsa_circ_0000284 as the endogenous control.

** symbolizes P < 0.01, and *** symbolizes P < 0.001. Hsa_circ_0000284 were utilized as the endogenous controls.


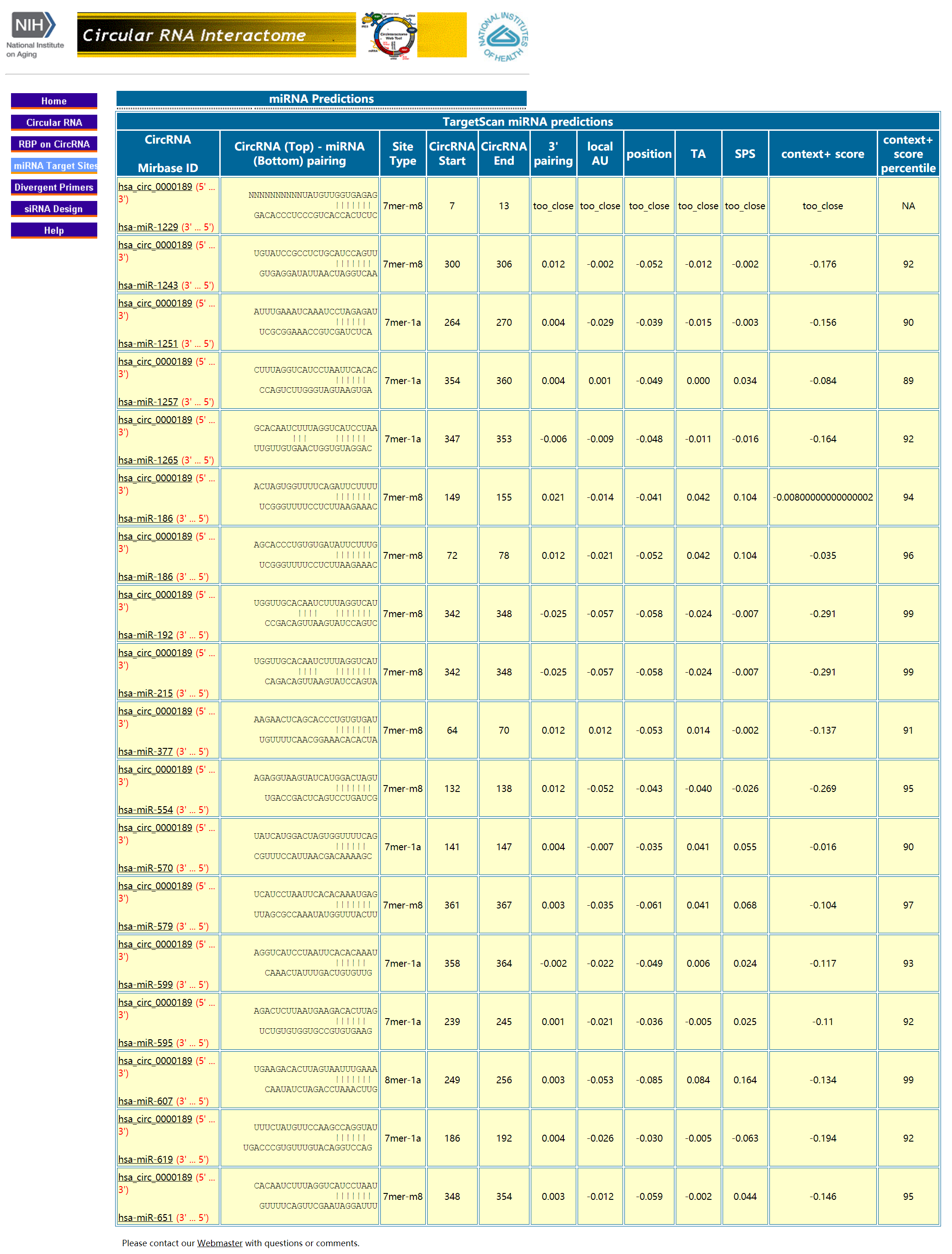


**Supplementary Figure 3. The** **potential targets of circ_0000189 predicted by Circinteractome database.**


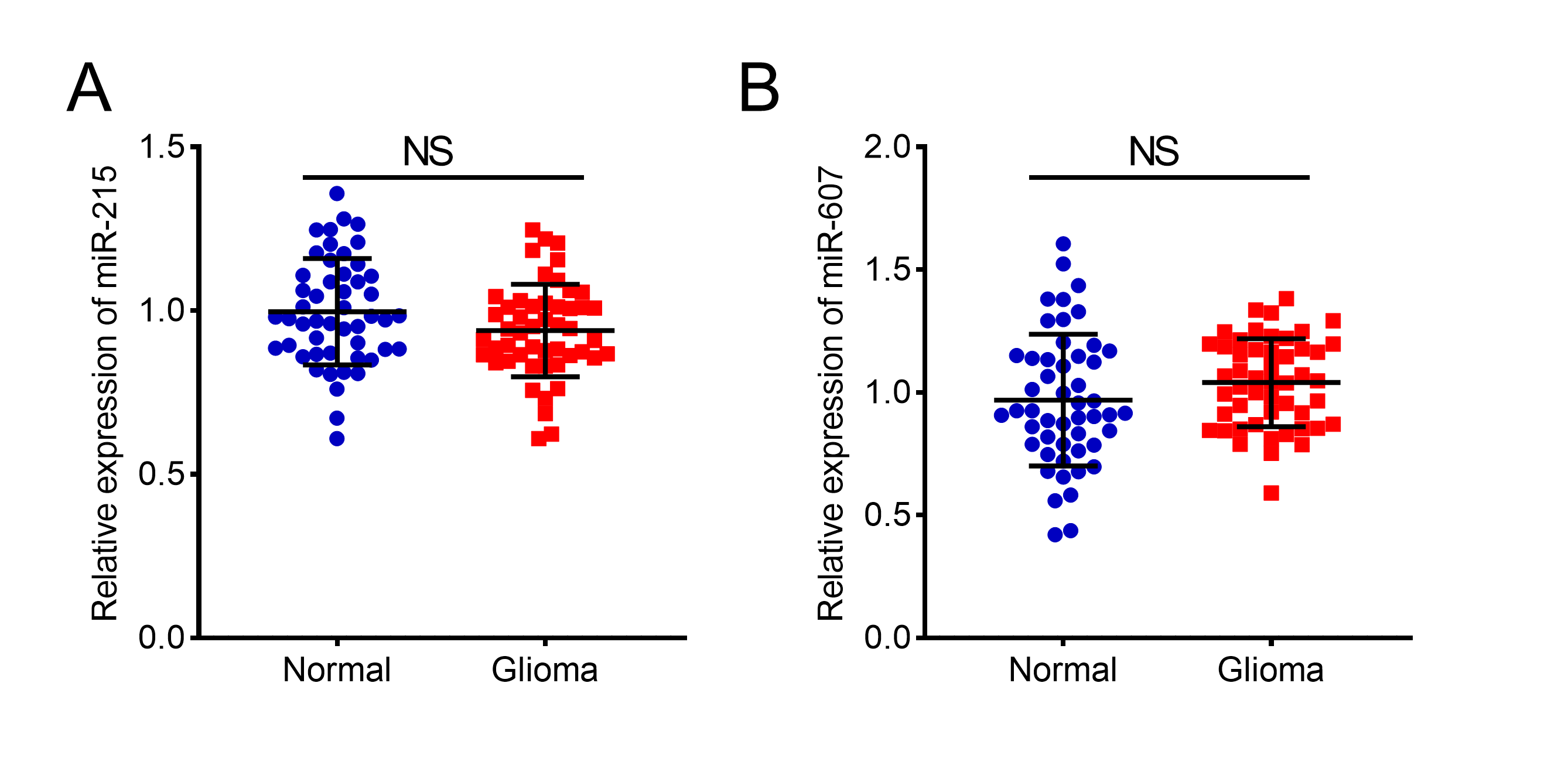


**Supplementary Figure 4. the expressions of miR-215 and miR-607 in normal tissues and glioma tissues was detected by qRT-PCR.**

| **Supplementary Table 1. The potential targets of miR-192-5p predicted by TargetScan database.** |
| --- |
| ABHD2 AC068987.1 ACTBL2 ACVR2A ACVR2B AL354993.1 ALCAM ALG6 ALKBH8 ALX1 AMER1 ANAPC16 ANKRD44 AP3M2 AP5M1 APTX ARFGEF1 ARHGAP19 ARHGAP36 ARHGEF39 ARL4C ASB6 ASXL2 ATG2A ATG7 ATP6V1C2 B3GALNT2 B4GALT2 BHLHE22 BLCAP C4orf46 C5orf27 CACNA1I CBL CCDC152 CCDC171 CCNT2 CDC6 CDON CENPP CHD7 CLSTN1 CLVS2 COL5A1 CPEB4 CREB5 CTCF CTH CTNNBIP1 CXCR5 DBT DCC DCK DDX49 DDX50 DIAPH1 DIAPH2 DICER1 DKFZP779L1853 DLG5 DNAH17-AS1 DNAJC19 DNMT3A DPP10 DST DYRK3 EIF1 EIF5A2 ELP4 EMC7 ENC1 ENY2 EREG FABP3 FAM167A FAM229B FAXC FGFR1OP FNDC3B FOXN1 FRK FRMD4B FTO GABPB2 GALNTL6 GAPVD1 GDF11 GLP1R GMEB1 GPR126 GPR22 GRIN2B H3F3B HIGD1A IER5 IGDCC3 IL6ST INTS8 KCNA7 KCNJ6 KCNK1 KCNK3 KIF1B KLHL15 KPNA4 KPNA6 KRBA2 LDB3 LIMS1 LMTK2 LPAR4 LRIG2 MAPK1 MCTS1 MFAP3 MIPOL1 MMP16 MSN MTMR4 MYLK MYO9A NAA50 NCOA3 NDUFB9 NFAT5 NIPAL1 NIPBL NKAIN2 NKX2-5 NR6A1 OLIG3 OSBPL10 PAPLN PARP8 PCDH17 PCK2 PCNP PDHB PDP1 PHTF2 PKP4 PLEKHO1 PLXNB2 PPP1R1C PPP1R3D PRKACB PRKAR1A PRKG1 PRPF31 PTCHD1 PTPRT RAB2A RAB32 RAD51L3-RFFL RAD54B RAP1GAP2 RB1 RFFL RFX6 RICTOR RNF217 RPAP2 RUNX1 RUNX1T1 S1PR1 SCARF1 SCN1A SERINC4 SH3RF3 SLC11A2 SLC23A3 SLC25A11 SLC39A6 SLC5A3 SLC9B2 SMC5 SNX33 SOAT1 SOGA3 SRGAP3 SRPX2 SRSF6 SYT6 TAOK1 TCEB3 TCTEX1D1 TDG TMTC3 TOR1A TOR1AIP1 TRERF1 TRIM44 TRPM7 TSHZ2 TYMS TYRP1 UBE2QL1 UBE2V2 USP1 VPS53 WDR44 WDR7 WNK1 WSCD2 WWC2 XIAP XPO4 YY1 ZBTB20 ZBTB34 ZC3HAV1 **ZEB2** ZFHX3 ZFP36L1 ZNF280C ZNF366 ZNF536 ZPBP2 |
